# Starch isolated from different hulless barley cultivars differs in their chemical and structural characteristics

**DOI:** 10.1002/fsn3.1084

**Published:** 2019-06-26

**Authors:** Liang Li, Zhendong Liu, Tieqiao Wang, Bo Wang, Wenhui Zhang, Guanghuan Li, Zhaoling Guo, Yongxian Zhang, Bei Xue, Zhang Luo

**Affiliations:** ^1^ Food Science College Tibet Agriculture & Animal Husbandry University Nyingchi China; ^2^ TAAHC‐SWU Medicinal Plants Joint Research and Development Centre Tibet Agricultural and Animal Husbandry College Nyingchi China; ^3^ Institute of Agriculture Products Development and Food Science Research of Tibet Academy of Agriculture and Animal Science Lhasa China

**Keywords:** differential scanning calorimetry, hulless barley starch, pasting properties

## Abstract

This study aimed to isolate starch and evaluate its chemical and structural characteristics from six Chinese hulless barley (HB) cultivars. Starch isolated from naked barley displays A‐type crystalline packing and a regular granular shape. We measured peak viscosity values ranging from 237 to 264 cP, trough viscosity values from 305 to 380 cP, breakdown values from 390 to 535 cP, final viscosities from 357 to 523 cP, setback values from 245 to 354 cP and 383 to 460 cP, peak times from 5.53 to 5.73 min, and pasting temperatures from 93.10 to 94.65°C by RVA. Transition temperatures (*T*_0_, *T*_p_, and *T*_c_), gelatinization temperature ranges (Δ*T*_r_), and enthalpies of gelatinization (Δ*H*) were measured on a differential scanning calorimeter analyzer (DSC) and ranged from 57.81 to 61.25°C, 64.36 to 67.57°C, 82.03 to 84.70°C, and 21.52 to 26.89°C and 7.14 to 10.66 J/g, respectively. The varying chemical and structural characteristics of HB starch isolated from different cultivars suggested the potential for broader applications of the cereal.

## 1 INTRODUCTION

Barley (also known as *Hordeum vulgare* L.) represents the 4th most valued cereal grain after rice, wheat, and corn and is classed as hulled or hulless according to the existence of husk (International Barley Genome Sequencing Consortium et al., 2012; Tang, Ando, Watanabe, Takeda, & Mitsunaga, 2001; Wang et al., 2011). Both barley types are adaptable crops highlighted by their higher tolerance to soil salt content compared to wheat (Cozzolino, Roumeliotis, & Eglinton, 2013). Barley is generally a summer crop but is grown in the winter in tropical climates (International Barley Genome Sequencing Consortium et al., 2012). Hulless barley (HB) originates from the Qinghai–Tibet Plateau of China (Wang et al., 2011), where it has been a foodstuff in Tibet since the 5th century. HB has the nutritive value of wheat and hulled barley, can be metabolized, and has a high protein and low fiber content (Rezaei, Dehghan, & Ayatollahy, 2008). HB is used in animal feed in areas of reduced rainfall but has attracted interest among researchers because of its solubility, high malt quality, ease of preparation, and β‐glucan and arabinoxylan content (Guo et al., 2018; Lin et al., 2018; Wang et al., 2011).

Hulless barley consists of ~80% complex carbohydrates, 11.5%–14.2% protein, 4.7%–6.8% lipids, 1.8%–2.4% ash, and 3.7%–7.7% β‐glucans. Starch is its major constituent accounting for 56%–75% of kernel weight (Li, Vasanthan, Rossnagel, Hoover, & Starch, 2001). The amylose percent of the starch of HB ranges from 0% to 40% dependent on the variety (Bhatty, 1997; Li et al., 2001; Zheng, Han, & Bhatty, 1998). The chemical and functional characteristics of each type are dependent on the amylose content, amylopectin content, the ratio of these two components, and granule structure. It has been shown that HB starch has a bimodal granule distribution and large amylose/amylopectin molecules and that its amylopectin is long‐chained (Bhatty, 1997; Li, Vasanthan, Hoover, & Rossnagel, 2004; Li et al., 2001; Suh, Verhoeven, Denyer, & Jane, 2004; Zheng et al., 1998). HB starch ports are thought to be few in number, and its related varieties are limited. Further knowledge of the starch characteristics is therefore required.

To date, the structural and chemical properties of HB starch have not been compared in different Chinese samples. Here, we studied the morphology, heat, and gelatinization characteristics of six HB starch samples isolated from major producing areas in China. X‐ray powder diffraction (XRD) and scanning electron microscopy (SEM) were used to study particle characteristics, paste properties were measured by RVA, and thermal properties were evaluated by differential scanning calorimeter (DSC). The currently selected variety is a newly developed variety that has a large cultivation volume, high yield, and strong adaptability in Tibet in the past 10 years, and basically covers the main varieties of the current barley in Tibet. Among them, black barley is a newly developed variety containing anthocyanins in Tibet, and the others are white barley. It is worth noting that the starch of the new main cultivar has not been studied and compared, and it has research value, and it is easy to collect in Tibet, which is conducive to experimentation. Moreover, the differences in the nature of these six species are also obvious. The results of this study have great guiding significance for the deep processing of barley. After studying and comparing their properties, they can have a clear understanding of the advantages and disadvantages of each species and use their unique advantages to apply to their suitable fields.

## 2 METHODS

### 2.1 Sample preparation

Seeds of HB (including Dongqing 18, Zangqing 2000, Zangqing 25, Black HB, Dongqing 17, and Dongqing 11) were collected in 2018 from the major production areas of China by the Agricultural Research Institute of Tibet Academy of Agricultural and Animal Husbandry Sciences. All samples were obtained from a single field and harvested prior to analysis.

Hulless barley starch was obtained according to methods described by Bello‐Pérez group with minor modifications (Bello‐Pérez, Agama‐Acevedo, Zamudio‐Flores, Mendez‐Montealvo, & Rodriguez‐Ambriz, 2010; Li et al., 2014). Briefly, grains were washed in water and then immersed in 0.1% anhydrous sodium sulfite (solution/grain ratio = 6:1) at 20°C for 48 hr while stirring. After removing the soaking liquid, the grains were washed in water. Samples were homogenized in a pulping machine, following which the slurry was passed through mesh nylon. Residues were washed in water to release all the starch, and remnants on the cloth were discarded. Samples were centrifuged at 2504 *g* for 12 min, and the supernatants were discarded. The upper precipitate (pigment layer) was removed, and the starch was separated by resuspending in water. Samples were centrifuged, the starch paste was washed in ethanol (95%), and samples were centrifuged three times. Supernatants were discarded, and the starch was oven‐dried at 50°C for 12 hr.

### 2.2 Characterization and analysis

#### 2.2.1 Starch granule morphology

Morphology was assessed by scanning electron microscope (SEM; Li et al., 2014). Starch samples were prepared in aluminum sample holders sealed with carbon tape metalized with gold to promote the reflection of electron beams. The samples were imaged on a ZEISS EVO 18 SEM (under a vacuum of 1.5 × 10^−3^ Pa, magnification = 500 and 2,000×, acceleration voltage = 3.0 kV).

#### 2.2.2 Granule size

The sample was stirred in ethanol at 626 *g* (Mohapatra et al., 2018), and then, the particle size was measured using a laser diffraction particle size analyzer (Mastersizer 2000).

#### 2.2.3 Amylose content

The amount of amylose in various starch was measured using colorimetric assays described by Zavareze group (Bruni, Oliveira, Halal, & Flores, 2017). Briefly, 100 mg of the samples was added to 100‐ml flasks and mixed with 96° GL ethanol and 1 mol/L NaOH. The samples were incubated at 100°C for 10 min in a water bath and cooled for 30 min, and distilled water was added. Aliquots (5 ml) were transferred to 100‐ml flasks containing acetic acid (1 ml, 1 mol/L) and iodine (2 ml of 2% [w/v]) and filled with water. Data were calculated based on the standard curves of pure amylose (Sigma). The absorbance was measured at 610 nm.

#### 2.2.4 Crystallinity

The crystallinity of the starch was analyzed by obtaining an X‐ray diffraction pattern on a D8 Advance XRD (Bruker AXS). The crystallinity was evaluated on MDI Jade 6 software to fit the measured XRD peak. R% crystallinity is used to measure the ratio of peak area, background area, and peak area (Liu et al., 2017).

#### 2.2.5 Viscosity

In a typical procedure, 2.0 g of starch is suspended in 28 g of water, incubated at 30°C for 2 min, incubated at 95°C for 8 min, and cooled at 50°C for 8 min. Adhesive properties were evaluated in the literature using RVA‐3C (Newport Scientific; Blazek & Copeland, 2008).

#### 2.2.6 Thermal properties

Thermal analysis of the starch was performed according to the method described by the Zhu. F team (Zhu & Xie, 2018). The enthalpy and peak gelation based on the starting temperature were measured. Two milligram of samples was weighed in an aluminum capsule, and 7 μl of water was added; then, the sealed capsule was allowed to stand for 30 min, followed by a PerkinElmer DSC 8500 at 20–120°C/min flow rate for thermal analysis (under a nitrogen atmosphere; scanning rate of 50 ml/min; Zhu & Xie, 2018).

### 2.3 Statistical analysis

The data obtained were the average values of the six HB varieties (*n* = 3). A two‐way ANOVA test was used for statistical analysis using ANOVA and Tukey's test. Analysis was performed by SPSS 16.0 statistical software. *p* < 0.05 indicates that the difference is statistically significant.

## 3 RESULTS AND DISCUSSION

### 3.1 Characterization of starch granule

**Figure 1:**
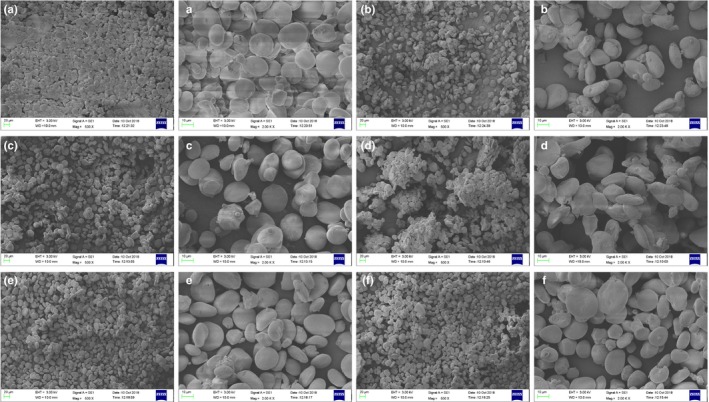
Varieties of hulless barley (HB) starch assessed: Dongqing 18: A (500×), a (2,000×); Zangqing 2000: B (500×), b (2,000×); Zangqing 25: C (500×), c (2,000×); Black HB: D (500×), d (2,000×); Dongqing 17: E (500×), e (2,000×); and Dongqing 11: F (500×), f (2,000×)

From the SEM results in Figure 1, it can be observed that the starch granules are oval, spherical, and polygonal. Moreover, the particle size of the particles ranges from 10 to 20 μm. It can be seen from Table 1 that the average particle diameter of the starch is from 18.99 to 23.17 μm. Among them, the starch having the smallest particle diameter is Dongqing 11, and the average particle diameter is 18.99 μm. The larger particle sizes are Nakano blue 25, Black HB, and Dongqing 18, and the average particle diameters are 20.33, 22.51, and 23.17 μm, respectively. In particular, it was found from the comparison of the overall morphology that the starch of the Black HB variety showed a state of particle agglomeration. On the contrary, the holly 18 was scattered and evenly distributed, and it was not easy to agglomerate. It is speculated that this result is related to the viscosity of the starch.

**Table 1:** Granule size and amylose % of starch obtained from HB cultivars

Starch	D (3,2) b	D (4,3) b	d (0.1) b	d (0.5) b	d (0.9) b	Amylose %
Dongqing 18	13.55 ± 0.01a	23.17 ± 0.01a	7.27 ± 0.01a	19.42 ± 0.01d	44.69 ± 0.01f	13.11 ± 0.03c
Zangqing 2000	14.54 ± 0.01c	19.18 ± 0.02a	10.66 ± 0.02d	18.94 ± 0.01b	28.66 ± 0.01a	15.72 ± 0.04f
Zangqing 25	15.54 ± 0.01f	20.33 ± 0.01a	11.56 ± 0.01f	19.56 ± 0.01e	30.12 ± 0.01d	15.18 ± 0.02e
Black HB	15.28 ± 0.02e	22.51 ± 0.01a	10.16 ± 0.01c	21.29 ± 0.01f	36.52 ± 0.01e	12.71 ± 0.03a
Dongqing 17	14.83 ± 0.01d	20.00 ± 0.01a	10.97 ± 0.02e	19.06 ± 0.02c	29.79 ± 0.01c	12.88 ± 0.05b
Dongqing 11	13.62 ± 0.01b	18.99 ± 0.01a	9.35 ± 0.01b	18.49 ± 0.01a	29.45 ± 0.01b	13.42 ± 0.01d

Note

All values are the means of triplicate determinations ± *SD*. The means within columns with different letters are significantly different (*p* < 0.05). b: d (0.1), d (0.5), and d (0.9) = granule sizes at which 10, 50, and 90% of granule volume are smaller, respectively; D (3,2) = surface‐area‐weighted mean diameter; D (4,3) = volume‐weighted mean diameter.

### 3.2 Evaluation of amylose

As can be seen in Table 1, the amylose content of the six HB varieties was 12.71%–15.72%. Among them, Zangqing 2000 has the highest amylose content, while Black HB has the lowest content. It has been reported that extensive variation in amylose is influenced by the type of variety and method of determination. The ratio of amylose affects starch paste viscosity and amylose content affects functional/physicochemical properties due to degradation (Blazek & Copeland, 2008; Kossmann & Lloyd, 2000; Uarrota et al., 2013). It is worth noting that many human nutrition research institutes abroad have done many experiments to confirm the healthcare value of amylose. High‐amylose foods are ideal for diabetics. High‐amylose starch is also an ideal food for patients with gallstones and hypertension and has the effect of preventing gallstone formation and lowering blood cholesterol. Amylose has stronger tensile strength and good formability, which can increase the brittleness and strength of the product. Therefore, Zangqing 2000 has a high amylose content, which may play a huge application value in the field of food production, processing, and even packaging materials.

### 3.3 Crystallinity assessment

**Figure 2:**
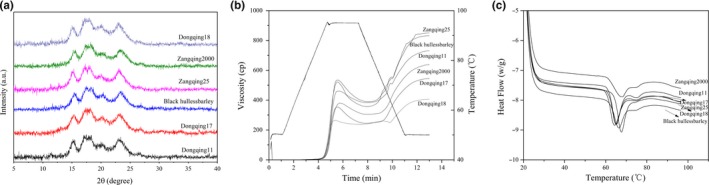
X‐ray diffraction spectra of HB starch (A); RVA profile of the isolated starch granules (B); DSC thermographs of the starch samples (C)

As can be seen from Figure 2, the six starch samples showed a similar XRD pattern at 15° and 23° 2θ, and a doublet of 17° and 18° 2θ, with the type A crystallinity observed in the grain starch. The peak positions are consistent. Subsequently, the crystallinity of different starches is also summarized in Table 2. It can be found that Dongqing 18 starch has the highest crystallinity (31.06%) and Black HB has the lowest (11.81%). In particular, comparing the amylose content, it was found that there was no significant correlation between crystallinity and amylose content, and amylopectin chain lengths in different starches were different. Amylopectin is generally considered to be the determinant of starch crystallinity, while the presence of amylose disrupts the crystalline packing of amylopectin chains (Bhatty, 1997; Lopez‐Rubio, Flanagan, Gilbert, & Gidley, 2008).

**Table 2:** XRD data of starch samples isolated from the HB cultivars

Varieties	Diffraction peaks at 2θ values (°)/day spacing (Å)					Crystallinity (%)
	15°	17°	18°	20°	23°	
Dongqing 18	15.00 (5.90)	17.24 (5.14)	18.02 (4.92)	20.12 (4.41)	23.10 (3.85)	31.06 ± 1.46d
Zangqing 2000	15.64 (5.66)	17.20 (5.15)	18.15 (4.88)	20.06 (4.42)	23.30 (3.81)	17.38 ± 0.65b
Zangqing 25	15.14 (5.85)	17.04 (5.20)	18.09 (4.90)	20.03 (4.43)	23.20 (3.83)	20.37 ± 0.87bc
Black HB	15.20 (5.82)	17.44 (5.08)	17.44 (5.08)	20.42 (4.35)	23.36 (3.80)	11.81 ± 1.26a
Dongqing 17	15.34 (5.77)	17.54 (5.05)	18.28 (4.84)	20.31 (4.37)	23.34 (3.81)	12.66 ± 3.57a
Dongqing 11	15.42 (5.74)	17.07 (5.19)	18.30 (4.84)	20.07 (4.42)	22.96 (3.87)	22.99 ± 2.63c

Note

All values are the means of triplicate determinations ± *SD*. The means within columns with different letters are significantly different (*p* < 0.05).

### 3.4 Pasting characteristics

Characteristics of the starch samples are shown in Table 3, and the pasting curve is shown in Figure 2. Peak viscosity ranged from 237 to 264 cP, trough viscosity ranged from 305 to 380 cP, breakdown ranged from 390 to 535 cP, final viscosity ranged from 357 to 523 cP, setback values ranged from 245 to 354 cP and 383 to 460 cP, peak times ranged from 5.53 to 5.73 min, and passing temperature ranged from 93.10 to 94.65°C across the samples. Zangqing 25 displayed the highest peak (535 cP), trough (390 cP), and final viscosity (851 cP), while Dongqing 18 displayed the lowest peak (264 cP), trough (237 cP), breakdown (27 cP), final viscosity (406 cP), and setback viscosity (169 cP). Peak viscosity is indicative of granule swelling prior to breakdown (Bhatty, 1997; Lopez‐Rubio et al., 2008). Breakdown viscosity is the difference between peak and trough viscosity and represents the degree of granule disintegration, swollen granule disruption, and amylose release during breakdown. Dongqing 18 had the minimum peak viscosity and breakdown values suggestive of strong cohesive force within its starch granules, thermal stability, and low levels of degradation (Kaur, Singh, Ezekiel, & Guraya, 2007).

**Table 3:** Pasting profiles of HB starch samples

Varieties	PV (cP)	TV (cP)	BD (cP)	FV (cP)	SB (cP)	PT (min)	GT (°C)
Dongqing 18	264 ± 18a	237 ± 46a	27 ± 2a	406 ± 18a	169 ± 43a	5.53 ± 0.06b	93.80 ± 0.31ab
Zangqing 2000	380 ± 42b	305 ± 38ab	75 ± 3b	638 ± 21c	333 ± 46b	5.73 ± 0.04c	95.50 ± 1.06c
Zangqing 25	535 ± 35d	390 ± 43c	145 ± 1d	851 ± 33e	461 ± 24c	5.53 ± 0.07b	93.10 ± 0.84a
Black HB	523 ± 29d	357 ± 56bc	166 ± 2e	831 ± 28e	474 ± 62c	5.53 ± 0.03b	93.10 ± 0.62a
Dongqing 17	354 ± 13b	245 ± 20a	109 ± 3c	545 ± 42b	300 ± 25b	5.53 ± 0.02b	94.65 ± 0.44bc
Dongqing 11	460 ± 6c	383 ± 34c	77 ± 3b	731 ± 34d	348 ± 53b	3.40 ± 0.01a	93.10 ± 0.51a

Note

All values are the means of triplicate determinations ± *SD*. The means within columns with different letters are significantly different (*p* < 0.05).

Abbreviations: BD, breakdown; FV, final viscosity; GT, pasting temperature; PT, peak time; PV, peak viscosity; SB, setback; TV, trough viscosity.

Importantly, the final viscosity is a measure of the stability of cooked paste, which tends to increase with cooling owing to the accumulation of resident amylose. The setback viscosity is a measure of the final viscosity and the peak viscosity, the difference being the measure of the viscosity of the heated starch paste and the rate of degradation after gelatinization during cooling. The lowest setback viscosity was for Dongqing 18 granules that had a reduced tendency to degrade. Zangqing 25, Black HB, and Dongqing 11 had the lowest pasting temperatures (93.10°C), while Zangqing 2000 had the highest (95.50°C). The majority of HB starch samples had a pasting temperature of ~93.87°C.

### 3.5 DSC

Figure 2 shows the DSC thermographs of the HB starch samples. The onset gelatinization temperature (*T*_0_), conclusion temperature (*T*_c_), peak temperature (*T*_p_), gelatinization temperature range (Δ*T*_r_), and enthalpies of gelatinization (Δ*H*) significantly differed among samples. The transition temperature (*T*_0_, *T*_p_, and *T*_c_), Δ*T*_r_, and Δ*H* ranged from 57.81 to 61.25°C, 64.36 to 67.57°C, 82.03 to 84.70°C, and 21.52 to 26.89°C and 7.14 to 10.66 J/g, respectively. Dongqing 17 showed the lowest onset and conclusion temperatures. The maximum onset temperature was Dongqing 18. Higher gelatinization temperatures indicate a greater energy requirement for starch gelatinization. Gelatinization temperatures among the starch samples differed according to crystallinity, granule shape and size, % amylose, amylopectin length, and the arrangement of starch fractions within granules. Dongqing 17 displayed the highest Δ*T*_r_ value (26.89°C), with Dongqing 18 starch showing the lowest (21.52°C) (Table 4; Kaur et al., 2007; Kaur, Singh, Ezekiel, & Sodhi, 2009; Kaur & Singh, 2005; Zhou, Robards, Helliwell, & Blanchard, 2007). The Δ*T*_r_ range indicated the presence of crystals of varying stability within the granule. A higher Δ*T*_r_ occurs due to higher crystallinity. The difference in Δ*T*_r_ among the starch samples is owing to the presence of crystalline regions of varying strength. Δ*H* values for the HB varieties ranged from 7.14 to 10.66 J/g; the lowest is observed for Zangqing 2000 and the highest for Dongqing 17. Δ*H* is caused by the breakage of double helices and long‐range disruption in crystallinity. The elevated Δ*H* of Dongqing 17 starch infers enhanced double helical disruption during gelatinization compared to other HB starches. This suggested a greater energy requirement was required to disrupt intermolecular interactions in the starch granules during gelatinization (Kaur et al., 2009; Miao, Zhang, & Jiang, 2009).

**Table 4:** Starch thermal characteristics

Starch	*T*_0_ (°C)	*T*_p_ (°C)	*T*_c_ (°C)	Δ*T*_r_ (°C)	∆*H* (J/g)
Dongqing 18	61.25 ± 0.35d	67.57 ± 0.31d	82.77 ± 0.38ab	21.52 ± 0.41a	8.92 ± 0.16c
Zangqing 2000	59.40 ± 0.24c	67.51 ± 0.24d	82.60 ± 0.56ab	23.20 ± 0.62b	7.14 ± 0.05a
Zangqing 25	58.87 ± 0.62bc	65.13 ± 0.43b	82.03 ± 0.46a	23.16 ± 0.46b	8.66 ± 0.38bc
Black HB	58.65 ± 0.06b	66.34 ± 0.51c	82.94 ± 0.74ab	24.29 ± 0.29c	8.56 ± 0.11b
Dongqing 17	57.81 ± 0.41a	64.36 ± 0.31a	84.70 ± 0.51c	26.89 ± 0.35d	10.66 ± 0.16e
Dongqing 11	58.73 ± 0.22bc	65.12 ± 0.28b	83.36 ± 0.33b	24.63 ± 0.31c	9.84 ± 0.09d

Note

All values are the means of triplicate determinations ± *SD*. The means within columns with different letters are significantly different (*p* < 0.05).

Abbreviations: ∆*H*, enthalpy; *T*_0_, gelatinization temperature; *T*_c_, temperature at which gelatinization ceased; *T*_p_, endothermic peak temperature.

## 4 CONCLUSION

We demonstrate the diversity of HB starch from different cultivars regarding physicochemical and morphological, pasting, thermal, and structural properties. This suggests that starch suits an array of applications. The starch from HB cultivars has comparable crystalline packing and granule shape, but the degree of crystallinity significantly differs. Experimental results have shown that among the six species, the extraction rate of Dongqing 18 and Zangqing 2000 starch is high, which is most suitable for food applications.

Hulless barley starch has the following potential applications: firstly, convenient food is processed. General foods are expected to obtain highly gelled and unaged product slag. Highly gelatinized foods are soft, palatable, easy to rehydrate, and easily digested by amylase. Secondly, the modified starch is processed. The modified starch has the characteristics of good rehydration, high viscosity, and stable viscosity. It can be used as a binder, thickener, and sizing agent in food, aquatic feed, paper, textile, and other industries. Characteristically, the development of specialty drinks and food: The Qinghai–Tibet Plateau has a high climate and a cold climate, making people demand more wine. Green barley contains 10.1% protein, 1.8% fat, and 70% carbohydrates and is rich in minerals, amino acids, and vitamins the body needs. This is a good brewing material. It is rich in nutrients and has higher nutritional value than rice, corn, and common wheat. It has a large calorific value and is full of hunger and cold. It is easy to carry and suitable for the lives of herders. In addition, it is recommended to develop green barley food, including barley biscuits, barley cakes, barley noodles, and other products, in view of high barley protein content, low gluten content, high amylopectin content, high material viscosity, reasonable processing technology and formula design, optimized operating conditions, high‐quality breakfast foods, snack foods, and nutritional health care for people with high blood lipids.

However, further studies are required to assess the desirable characteristics and commercial application of each starch through the analysis of their structural, physical, and functional properties. Studies investigating the impact of modifications on the structural and physiochemical properties of HB starch sampled from the different cultivars are also warranted.

## CONFLICT OF INTEREST

The authors declare that they do not have any conflict of interest.

## ETHICAL APPROVAL

This study does not involve any human or animal testing.

## INFORMED CONSENT

Written informed consent was obtained from all study participants.

## References

[fsn31084-bib-0001] Bello‐Pérez, L. A. , Agama‐Acevedo, E. , Zamudio‐Flores, P. B. , Mendez‐Montealvo, G. , & Rodriguez‐Ambriz, S. L. (2010). Effect of low and high acetylation degree in the morphological, physicochemical and structural characteristics of barley starch. LWT ‐ Food Science and Technology, 43, 1434–1440. 10.1016/j.lwt.2010.04.003

[fsn31084-bib-0002] Bhatty, R. S. (1997). Milling of regular and waxy starch hull‐less barleys for the production of bran and flour. Cereal Chemistry Journal, 74, 693–699. 10.1094/CCHEM.1997.74.6.693

[fsn31084-bib-0003] Blazek, J. , & Copeland, L. (2008). Pasting and swelling properties of wheat flour and starch in relation to amylose content. Carbohydrate Polymers, 71, 380–387. 10.1016/j.carbpol.2007.06.010

[fsn31084-bib-0004] Bruni, G. P. , de Oliveira, J. P. , El Halal, S. L. M. , Flores, W. H. , Gundel, A. , de Miranda, M. Z. , … da Rosa Zavareze, E. (2017). Phosphorylated and cross‐linked wheat starches in the presence of polyethylene oxide and their application in biocomposite films. Starch ‐ Stärke, 70, 1700192.

[fsn31084-bib-0005] Cozzolino, D. , Roumeliotis, S. , & Eglinton, J. (2013). Relationships between starch pasting properties, free fatty acids and amylose content in barley. Food Research International, 51, 444–449. 10.1016/j.foodres.2013.01.030

[fsn31084-bib-0006] Guo, H. , Lin, S. , Lu, M. , Gong, J. D. B. , Wang, L. U. , Zhang, Q. , … Wu, D.‐T. (2018). Characterization, in vitro binding properties, and inhibitory activity on pancreatic lipase of β‐glucans from different Qingke (Tibetan hulless barley) cultivars. International Journal of Biological Macromolecules, 120, 2517–2522. 10.1016/j.ijbiomac.2018.09.023 30195000

[fsn31084-bib-0007] Kaur, A. , Singh, N. , Ezekiel, R. , & Guraya, H. S. (2007). Physicochemical, thermal and pasting properties of starches separated from different potato cultivars grown at different locations. Food Chemistry, 101, 643–651. 10.1016/j.foodchem.2006.01.054

[fsn31084-bib-0008] Kaur, A. , Singh, N. , Ezekiel, R. , & Sodhi, N. S. (2009). Properties of starches separated from potatoes stored under different conditions. Food Chemistry, 114, 1396–1404. 10.1016/j.foodchem.2008.11.025

[fsn31084-bib-0009] Kaur, M. , & Singh, N. (2005). Studies on functional, thermal and pasting properties of flours from different chickpea (*Cicer arietinum* L.) cultivars. Food Chemistry, 91, 403–411. 10.1016/j.foodchem.2004.06.015

[fsn31084-bib-0010] Kossmann, J. , & Lloyd, J. (2000). Understanding and influencing starch biochemistry. Critical Reviews in Plant Sciences, 19, 171–226. 10.1016/S0735-2689(00)80002-7 10907795

[fsn31084-bib-0011] Li, J. H. , Vasanthan, T. , Hoover, R. , Rossnagel, B. G. (2004). Starch from hull‐less barley: IV. Morphological and structural changes in waxy, normal and high‐amylose starch granules during heating. Food Research International, 37, 417–428. 10.1016/S0963-9969(04)00046-8

[fsn31084-bib-0012] Li, J. H. , Vasanthan, T. , Rossnagel, B. , & Hoover, R. (2001). Starch, from hull‐less barley: I. Granule morphology, composition and amylopectin structure. Food Chemistry, 74, 395–405. 10.1016/S0308-8146(01)00246-1

[fsn31084-bib-0013] Li, W. , Xiao, X. , Zhang, W. , Zheng, J. , Luo, Q. , Ouyang, S. , & Zhang, G. (2014). Compositional, morphological, structural and physicochemical properties of starches from seven naked barley cultivars grown in China. Food Research International, 58, 7–14. 10.1016/j.foodres.2014.01.053

[fsn31084-bib-0014] Lin, S. , Guo, H. , Gong, J. D. B. , Lu, M. , Lu, M.‐Y. , Wang, L. U. , … Wu, D.‐T. (2018). Phenolic profiles, β‐glucan contents, and antioxidant capacities of colored Qingke (Tibetan hulless barley) cultivars. Journal of Cereal Science, 81, 69–75. 10.1016/j.jcs.2018.04.001

[fsn31084-bib-0015] Liu, M. , Wu, N.‐N. , Yu, G.‐P. , Zhai, X.‐T. , Chen, X. , Zhang, M. , … Tan, B. (2017). Physicochemical properties, structural properties, and in vitro digestibility of pea starch treated with high hydrostatic pressure. Starch ‐ Stärke, 70, 1700082.

[fsn31084-bib-0017] Lopez‐Rubio, A. , Flanagan, B. M. , Gilbert, E. P. , & Gidley, M. J. (2008). A novel approach for calculating starch crystallinity and its correlation with double helix content: A combined XRD and NMR study. Biopolymers, 89, 761–768. 10.1002/bip.21005 18428208

[fsn31084-bib-0018] International Barley Genome Sequencing Consortium , Mayer, K. F. X. , Waugh, R. , Brown, J. W. , Schulman, A. , Langridge, P. , … Stein, N. (2012). A physical, genetic and functional sequence assembly of the barley genome. Nature, 491, 711–716. 23075845 10.1038/nature11543

[fsn31084-bib-0019] Miao, M. , Zhang, T. , & Jiang, B. (2009). Characterisations of kabuli and desi chickpea starches cultivated in China. Food Chemistry, 113, 1025–1032. 10.1016/j.foodchem.2008.08.056

[fsn31084-bib-0020] Mohapatra, S. , Siddiqui, A. A. , Anwar, M. , Bhardwaj, N. , Akhter, S. , & Ahmad, F. J. (2018). Synthesis and characterization of novel carboxymethyl Assam Bora rice starch for the controlled release of cationic anticancer drug based on electrostatic interactions. An Official Journal of the American Association of Pharmaceutical Scientists, 19, 134–147. 10.1208/s12249-017-0824-z 28631252

[fsn31084-bib-0021] Rezaei, M. , Dehghan, M. , & Ayatollahy, M. (2008). Determination of metabolisable energy of five cultivars of hulless barley using adult leghorn cockerels. South African Journal of Animal Science, 38, 28–30.

[fsn31084-bib-0022] Suh, D. S. , Verhoeven, T. , Denyer, K. , & Jane, J. (2004). Characterization of Nubet and Franubet barley starches. Carbohydrate Polymers, 56, 85–93. 10.1016/j.carbpol.2003.12.005

[fsn31084-bib-0023] Tang, H. , Ando, H. , Watanabe, K. , Takeda, Y. , & Mitsunaga, T. (2001). Physicochemical properties and structure of large, medium and small granule starches in fractions of normal barley endosperm. Carbohydrate Research, 330, 241–248. 10.1016/S0008-6215(00)00292-5 11217977

[fsn31084-bib-0024] Uarrota, V. G. , Amante, E. R. , Demiate, I. M. , Vieira, F. , Delgadillo, I. , & Maraschin, M. (2013). Physicochemical, pasting properties of flours and starches of eight Brazilian maize landraces (*Zea mays* L.). Food Hydrocolloids, 30, 614–624. 10.1016/j.foodhyd.2012.08.005

[fsn31084-bib-0025] Wang, C.‐P. , Pan, Z.‐F. , Nima, Z.‐X. , Tang, Y.‐W. , Cai, P. , Liang, J.‐J. , … Yu, M.‐Q. (2011). Starch granule‐associated proteins of hull‐less barley (*Hordeum vulgare* L.) from the Qinghai‐Tibet Plateau in China. Journal of the Science of Food and Agriculture, 91, 616–624. 10.1002/jsfa.4223 21213217

[fsn31084-bib-0026] Zheng, G. H. , Han, H. L. , & Bhatty, R. S. (1998). Physicochemical properties of zero amylose hull‐less barley starch. Cereal Chemistry Journal, 75, 520–524. 10.1094/CCHEM.1998.75.4.520

[fsn31084-bib-0027] Zhou, Z. , Robards, K. , Helliwell, S. , & Blanchard, C. (2007). Effect of the addition of fatty acids on rice starch properties. Food Research International, 40, 209–214. 10.1016/j.foodres.2006.10.006

[fsn31084-bib-0028] Zhu, F. , & Xie, Q. (2018). Rheological and thermal properties in relation to molecular structure of New Zealand sweet potato starch. Food Hydrocolloids, 83, 165–172. 10.1016/j.foodhyd.2018.05.004

